# Liver Abscess Caused by *Pannonibacter phragmitetus*: Case Report and Literature Review

**DOI:** 10.3389/fmed.2017.00048

**Published:** 2017-04-25

**Authors:** Mingxi Wang, Xia Zhang, Tao Jiang, Shaohua Hu, Zhengjun Yi, Yajun Zhou, Desong Ming, Shicheng Chen

**Affiliations:** ^1^Yun Leung Laboratory for Molecular Diagnostics, School of Biomedical Sciences, Huaqiao University, Xiamen, Fujian, China; ^2^Department of Medical Laboratory, Institute of Nanomedicine Technology, Weifang Medical University, Weifang, Shandong, China; ^3^Department of Clinical Laboratory, Quanzhou First Hospital Affiliated to Fujian Medical University, Fujian, China; ^4^Department of Microbiology and Molecular Genetics, Michigan State University, East Lansing, MI, USA

**Keywords:** bacterial hepatic abscess, *Klebsiella pneumoniae*, *Pannonibacter phragmitetus*, polymicrobial infection, *Streptococcus oralis*

## Abstract

**Background:**

Bacterial hepatic abscess is a common occurrence in developing countries, which is mostly caused by *Klebsiella pneumoniae* and *Escherichia coli*. *Pannonibacter phragmitetus* is a Gram-negative alkali-tolerant bacillus that exists in the natural environment. Human infection by this bacterium is rare, with only four cases reported.

**Method:**

We presented one of these cases with a bacterial liver abscess by a polymicrobial infection involving *P. phragmitetus* and *Streptococcus oralis*, with *P. phragmitetus* being the predominate isolate.

**Result and discussion:**

Our strain of *P. phragmitetus* was resistant to more antibiotics than the other reported two strains. This case further verified the infectivity of *P. phragmitetus*.

## Background

*Pannonibacter phragmitetus* (*P. phragmitetus*) is a Gram-negative, motile rod that is frequently found to be star-shaped aggregates under microscopy (1). It is a facultative anaerobe and chemo-organotrophic (1). Before 2006, it was misidentified as *Achromobacter* groups B and E. Actually, *P. phragmitetus* and *Achromobacter* groups B and E are a single taxon (2). *P. phragmitetus* can survive in a high alkaline environment, such as Hungarian soda lake (1), and geothermal habitat (3, 4). It has the capacities of reducing hexavalent chromium (5–12). Isolation of this bacterium from a human sample was first reported in 1975 (13). So far, only four cases of infection by *P. phragmitetus* were reported: one case of replacement valve endocarditis (14), two cases of septicemia (15), and one case of recurrent septicemia (16). Here, we presented one case of liver abscess infected by *P. phragmitetus*, suspiciously combined with *Streptococcus oralis* (*S. oralis*).

## Case Report

**Figure 1:**
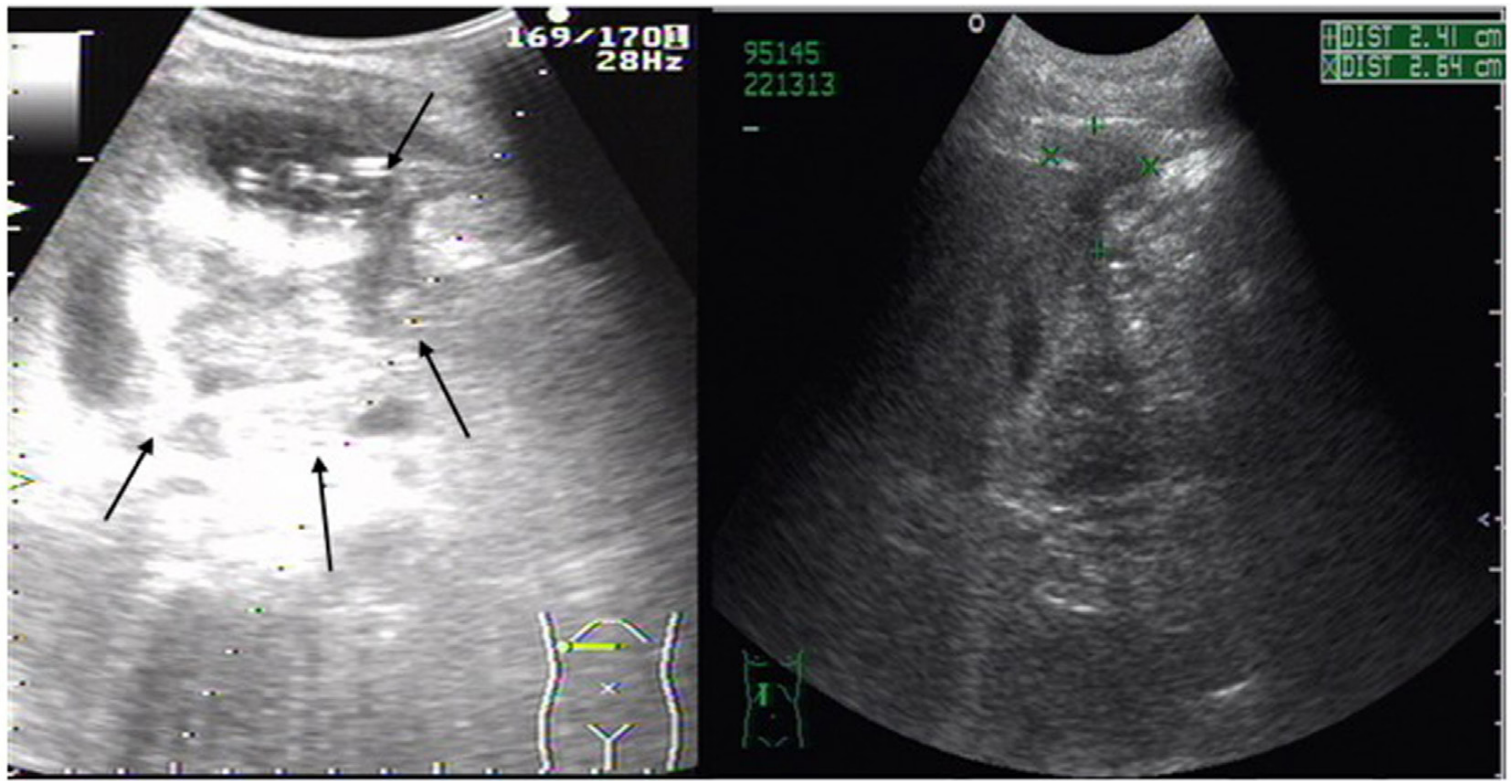
**Abdominal ultrasonography scan of liver abscess in right lobe of the liver**. Large (82 mm × 78 mm) multiple low-signal areas (shown by arrows) were detected before percutaneous abscess drainage. Its size (+, ×) was greatly reduced after percutaneous liver abscess drainage.

A 44-year-old male consumed more than 150 g of alcohol and smoked 20 cigarettes per day for 20 years. He was admitted to our hospital due to pain in the right upper abdomen for 14 days with normal body temperature. On a physical examination, he displayed right lateral upper abdominal tenderness. The liver edge was palpated 5 and 10 cm under the right costal margin and the xiphoid process, respectively, with surface protuberance and tenderness. Laboratory tests showed an elevated inflammatory reaction [white blood cell count (WBC) 1.7 × 10^10^/L, neutrophil 81.6%, C-reactive protein (CRP) level 325 mg/L] and anemia (RBC 3.28 × 10^12^/L, HGB 112 g/L). Abnormal liver function parameters were also noted [aspartate aminotransferase 47 IU/L, gamma-glutamyl transpeptidase 139 IU/L, and alkaline phosphatase 133 IU/L]. Ultrasonography detected a large abscess (82 mm × 78 mm) within the right liver lobe (Figure 1).

Intravenous injections of cefodizime sodium (1.5 g, b.i.d) and metronidazole (0.5 g, b.i.d) were administered daily from the first day after his admission to the end of the hospitalization (20 days). The patient underwent percutaneous abscess drainage using an 8.5-Fr catheter guided by ultrasonography on the fifth day of his hospitalization, which was left in place for 7 days. The patient fully recovered with the combination of antibiotic therapy and percutaneous abscess drainage. This was demonstrated by normal blood WBC count, CRP, negative blood, and abscess culture.

Using BD BACTEC 9240 auto blood culture system, *P. phragmitetus* strain 31801 was isolated from a blood sample collected within 24 h after his admission. The identity of *P. phragmitetus* strain 31801 was verified by 16S rRNA gene sequencing (GenBank number FJ882624.1) and whole genome sequencing strategy (accession number CP013068). The antimicrobial susceptibility test (AST) was conducted with the Kirby–Bauer disk diffusion test (OXOID, England) and BD Phoenix™ 100 Automated Microbiology System using NMIC/ID-109 identification/antibiotic susceptibility cards (Becton, Dickinson and Company). *P. phragmitetus* 31801 was sensitive to amikacin, imipenem, ceftazidime, cefepime, amoxicillin, piperacillin/tazobactam, gatifloxacin, and levofloxacin, while it was immediately sensitive to cefotaxime and ceftriaxone. It was resistant against gentamicin, tobramycin, piperacillin, trimethoprim/sulfamethoxazole, furazolidone, and tetracycline. Further studies showed that this strain was positive for extra-extended-spectrum β-lactamase as shown by the Kirby–Bauer test. It was shown to carry the ampicillin-inducible β-lactamase with some clones in the zone of CAZ + CA disk and ant (3″)-I that were verified by polymerase chain reaction. Using a blood agar plate, *S. oralis* was cultured from the liver abscess and collected on the fifth day after the patient’s admission when the abscess drainage was performed. It was unable to be recovered from the pus and blood sample collected later.

## Discussion

In China, the most common bacterial hepatic abscesses have been consistently caused by *Escherichia coli* and *Klebsiella* sp. (accounts for 83%) (17). However, other bacteria were also reported to be isolated from the liver abscess. A recent survey showed that Gram-negative bacteria *Enterobacter* spp. (9%), *Proteus* spp. (6%), and *Pseudomonas* spp. (5%) were factors that caused this disease, next to *E. coli* and *Klebsiella* sp. (17). Frequently, many cases were caused by Gram-positive bacteria, mainly including *Staphylococcus* spp. (13%), *Streptococcus* spp. (8%), and *Enterococcus* spp. (7%) (17). It is uncommon for polymicrobial infections in hepatic abscesses due to most of the cases being monomicrobial (18). In our patient, *S. oralis*, a Gram-positive bacterium, was isolated from the pus. It should be noted that liver abscess due to its infection is quite rare. It was only isolated from the pus samples from two liver abscess patients (19, 20). However, no report was found on the application of cefodizime for treating *S. oralis* infections, and it was known that *S. oralis* was not sensitive to metronidazole (19). Our patient was cured by metronidazole, cefodizime, and catheter drainage. Although we could not rule out the possibility that *S. oralis* was acquired from environmental contamination, we speculated that its contribution to this liver abscess was not significant.

Instead, *P. phragmitetus* 31801 may be the real pathogen of our patient seeing as it was isolated from the blood sample collected within 24 h after his admission to the hospital, and the liver abscess in our patient could be diagnosed by abdominal ultrasonography along with an elevated inflammatory reaction on the first day that he was admitted. The chance of contamination during collection of the blood is low. It was initially identified as *Achromobacter* species, with the use of BD Phoenix™ 100 Automated Microbiology System and verified by 16S rRNA gene sequencing. Although it was not isolated from abscess fluid, we believed that *P. phragmitetus* strain 31801 was the actual pathogen. We had misidentified *P. phragmitetus* strain 31801 as *Sphingomonas paucimobilis* during our interpretation of the result of BD Phoenix™ 100 Automated Microbiology System before it was verified by 16S rRNA gene sequencing. The infectivity of *P. phragmitetus* had been demonstrated even though only four cases of infection have been reported (13, 15, 16). The pathogenic mechanisms of *P. phragmitetus* were unknown; in fact, we found many virulence factors in *P. phragmitetus* when we analyzed the sequenced genome (unpublished). In an antimicrobial susceptibility test, this bacterium was resistant against several classifications of antibiotics mentioned above. Furthermore, it has extra-extended-spectrum β-lactamase, which was inducible and ant (3″)-I (21).

*Pannonibacter phragmitetus* 31801 was resistant to more antibiotics than the strain isolated by Jenks and Shaw, which was sensitive to gentamicin, trimethoprim/sulfamethoxazole, and tetracycline (16), and the strain isolated by McKinley et al., which was sensitive to trimethoprim/sulfamethoxazole and tetracycline (14), while *P. phragmitetus* 31801 was resistant against these three antibiotics: tobramycin, piperacillin, and furazolidone.

In conclusion, this case report raises awareness to the possibility of infectivity and multiple-drug resistance of *P. phragmitetus*.

## Ethics Statement

The written informed consent for the publication of this case report was obtained from the patient. This work was already approved by the Ethics Committee of Quanzhou First Hospital.

## Author Contributions

Conception and design of the work: MW, XZ, and YZ. Data collection: MW, XZ, TJ, SH, ZY, YZ, and DM. Data analysis and interpretation, manuscript writing, and critical revision of the article: MW, XZ, YZ, and SC. Approval of the final version of the article: MW, XZ, TJ, SH, ZY, YZ, DM, and SC.

## Conflict of Interest Statement

The authors declare that the research was conducted in the absence of any commercial or financial relationships that could be construed as a potential conflict of interest.
